# Specially Structured AgCuTi Foil Enables High-Strength and Defect-Free Brazing of Sapphire and Ti6Al4V Alloys: The Microstructure and Fracture Characteristics

**DOI:** 10.3390/ma17153812

**Published:** 2024-08-02

**Authors:** Shaohong Liu, Hairui Liu, Limin Zhou, Hao Cui, Manmen Liu, Li Chen, Ming Wen, Haigang Dong, Feng Liu, Wei Wang, Song Li

**Affiliations:** 1 Key Laboratory for Anisotropy and Texture of Materials (Ministry of Education), School of Materials Science and Engineering, Northeastern University, Shenyang 110819, China; lhairui@stumail.neu.edu.cn (H.L.);; 2 State Key Laboratory of Advanced Technologies for Comprehensive Utilization of Platinum Metals, Yunnan Precious Metals Laboratory Co., Ltd., Kunming 650106, China; 3 Sino-Platinum Metals Semiconductor Materials (Yunnan) Co., Ltd., Kunming 650106, China

**Keywords:** dissimilar joining, metal–ceramic bonding, active metal brazing, AgCuTi, sapphire/TC4 joint

## Abstract

A novel AgCuTi brazing foil with a unique microstructure was developed, which could achieve strong vacuum brazing of Ti6Al4V (TC4) and sapphire. The brazing foil was composed of Ag solid solution (Ag(s,s)), Cu solid solution (Cu(s,s)), and layered Ti-rich phases, and had a low liquidus temperature of 790 °C and a narrow melting range of 16 °C, facilitating the defect-free joining of TC4 and sapphire. The sapphire/TC4 joint fabricated by using this novel AgCuTi brazing foil exhibited an outstanding average shear strength of up to 132.2 MPa, which was the highest value ever reported. The sapphire/TC4 joint had a characteristic structure, featuring a brazing seam reinforced by TiCu particles and a thin Ti_3_(Cu,Al)_3_O reaction layer of about 1.3 μm. The fracture mechanism of the sapphire/TC4 joint was revealed. The crack originated at the brazing seam with TiCu particles, then propagated through the Ti_3_(Cu,Al)_3_O reaction layer, detached the reaction layer from the sapphire, and finally penetrated into the sapphire. This study offers valuable insights into the design of active brazing alloys and reliable metal–ceramic bonding.

## 1. Introduction

Many high-performance applications, such as window sealing, electronic packaging, electric vehicles, and renewable energy systems, rely on metal–ceramic joints [1,2,3,4]. However, the different thermal expansion and Young’s modulus of metal and ceramic make it difficult to obtain strong and stable bonds [5,6]. A possible solution is active metal brazing (AMB), which uses a braze alloy containing a reactive metal (usually titanium) that forms a chemical bond with the ceramic surface in a vacuum [7,8,9]. This allows direct brazing of metals to uncoated ceramics, saving time and cost [10,11]. To improve the performance of AMB metal–ceramic joints, two important aspects need to be considered: the optimization of active brazing alloys and the investigation of the fracture mechanism of metal–ceramic bonds. These are essential for the advancement of metal–ceramic bonding technology.

The performances of active brazing alloys are governed by their microstructure-dependent melting characteristics, such as solidus and liquidus temperatures and melting range [12,13]. A narrow melting range facilitates the quick melting and flow of the alloy at low temperatures, which is advantageous for brazing. On the other hand, a wide melting range can result in liquation, and the separation of low- and high-melting components during slow heating, which degrades the quality and reliability of the brazed joint by causing voids or poor wetting of the base materials. Moreover, the residual stress at the interface, which arises from cooling after brazing and can cause damage or fracture of the joint, is influenced by the melting characteristics of the brazing alloys [14]. Higher brazing temperatures lead to larger residual stresses [15]. Therefore, brazing alloys with low liquidus temperatures and narrow melting ranges are preferred. The most common active brazing alloys are derived from the 72Ag-28Cu eutectic alloy (with a eutectic temperature of 780 °C), which contains small amounts of titanium (the active element) in foil form [16]. Two typical AgCuTi brazing alloy foils are Ag-35.25Cu-1.75Ti (wt.%) and Ag-26.7Cu-4.5Ti (wt.%), commercially known as Cusil ABA^®^ and Ticusil^®^ (Morgan Advanced Materials, Windsor, UK), respectively [17,18,19,20]. The former is produced by melting, casting, and rolling, resulting in a microstructure with abundant Ti-Cu intermetallic compounds (IMCs), mainly TiCu_4_, which increase the liquidus temperature and the melting range to 815 °C and 35 °C, respectively [21]. The latter is fabricated by pack rolling AgCu/Ti/AgCu sheets, which have isolated Ti_3_Cu_4_ particles along the Ti/AgCu interfaces. Upon heating, more Ti_3_Cu_4_ forms due to the reaction between AgCu and Ti at the interfaces. The composition and uniformity of Ticusil^®^ are difficult to control, leading to a high liquidus temperature of up to 900 °C and a wide melting range of 120 °C [22]. Hence, novel AgCuTi brazing alloys with lower liquidus temperatures and narrower melting ranges are required.

The fracture behavior of metal–ceramic bonds is complex and not well understood. It depends on the crack initiation and propagation modes, which vary according to different factors. The literature reports four main fracture modes: (1) brazing seam cracking due to incomplete fusion or IMC accumulation, which produces dimpled fracture surfaces [23,24]; (2) cohesive failure, where the ceramic breaks along a bowl-shaped region of high residual stress near the brazing interface, indicating large ceramic residual stresses [16]; (3) ceramic/reaction layer interface cracking, which can be either adhesive, with separation of the ceramic and the reaction layer, or mixed, with ceramic crack deflection and fracture [18]; (4) metal/IMC interface cracking, which is followed by crack deviation and ceramic intrusion, resulting in ceramic brittle fracture [5]. These modes are not mutually exclusive and may coexist or interact depending on the joint geometry and loading conditions. The fracture mechanisms of metal–ceramic bonds need to be further clarified to improve joint performance.

In this study, a novel AgCuTi brazing foil with a distinctive microstructure was fabricated by powder metallurgy. The brazing foil had a low liquidus temperature of 790 °C and a narrow melting range of 16 °C, both significantly lower than those of the existing AgCuTi brazing alloys. The microstructure of the brazing foil consisted of Ag solid solution (Ag(s,s)), Cu solid solution (Cu(s,s)), and layered Ti-rich phases, which differed markedly from the known AgCuTi brazing alloys. The brazing foil enabled a strong (up to 132.2 MPa) and defect-free sapphire/Ti6Al4V (TC4) joint by vacuum brazing. This work advances the design of high-performance active metal brazing foil and the metal–ceramic bonding.

## 2. Experimental Section

### 2.1. Synthesis of AgCuTi Brazing Foil

A powder metallurgy method was used to synthesize AgCuTi brazing foil with a nominal composition of 63Ag-35.25Cu-1.75Ti (wt.%). High-purity (>99.9%) Ag, Cu, and TiH_2_ powders with an average particle size of ~50 μm were obtained from Kunming Institute of Precious Metals, Kunming, China, and Aladdin Industrial Corporation, Shanghai, China, respectively. The powders were mixed homogeneously by ball-milling for 120 min in air using zirconia balls as the milling media and then cold-pressed at 300 MPa into green pellets of 32 mm × 12 mm × 5 mm. The green pellets were sintered in a vacuum of 10^−2^ Pa at 700 °C for 30 min under a uniaxial load of 35 MPa. The sintered compacts were cold rolled to obtain AgCuTi brazing foil with a thickness of approximately 150 μm.

### 2.2. Brazing Processes

**Figure 1:**
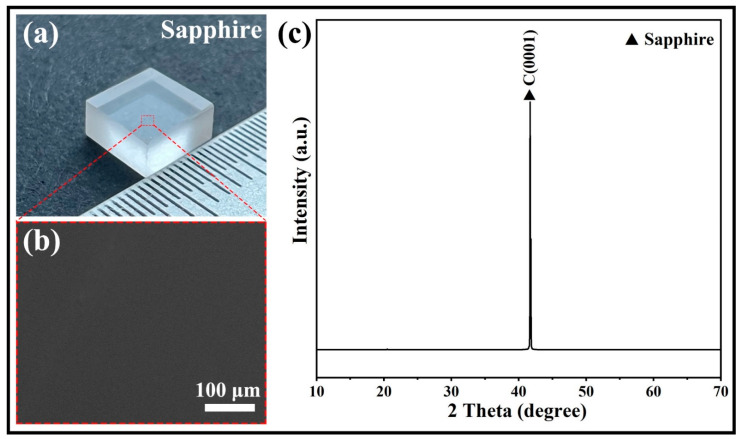
(**a**) Optical microscopy image of the sapphire plate showing its morphology and dimensions. (**b**) Scanning electron microscopy (SEM) image of the sapphire plate revealing its surface features and defects. (**c**) X-ray diffraction (XRD) pattern of the sapphire plate verifying its single-crystal structure.

**Figure 2:**
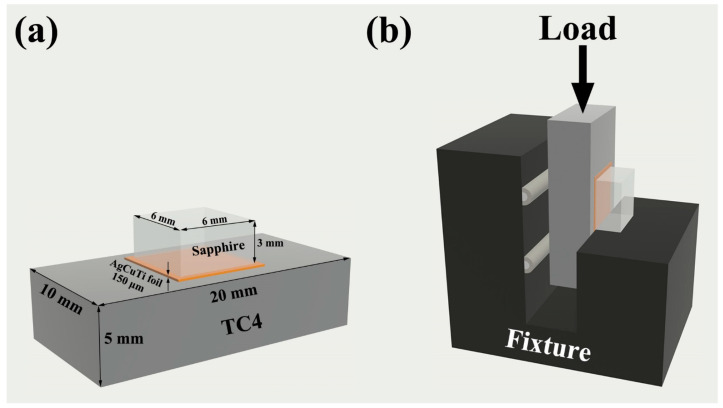
(**a**) A schematic diagram of the sandwich brazing configuration, showing the dimensions and materials of the components. (**b**) A schematic diagram of the shear strength testing device, showing the loading direction.

The brazing process aimed to join sapphire and TC4, a titanium alloy widely used in aerospace applications. The sapphire plates were transparent, single-crystalline, and had a C(0001) surface orientation, a purity of over 99.99%, and no visible defects (Figure 1). The dimensions of the sapphire plates were 6 mm × 6 mm × 3 mm. The dimensions of the TC4 were 20 mm × 10 mm × 5 mm. The 150 μm AgCuTi foil served as the brazing filler metal. Before brazing, the three materials were ultrasonically cleaned in acetone for 10 min and air-dried. The brazing assembly consisted of a sandwich configuration of sapphire plates, AgCuTi foil, and TC4 (Figure 2a). A load of about 1.2 kPa was applied to the assembly to ensure good contact between the materials. The brazing was performed at 800 °C for 10 min in a vacuum tube furnace with a pressure of 10^−3^ Pa. After brazing, the furnace was cooled down to room temperature at a rate of 5 °C min^−1^.

### 2.3. Characterization Techniques

The microstructure and elemental distribution of the samples were characterized by optical microscopy (OM, LEICA DMi8) (Wetzlar, Germany) and scanning electron microscopy (SEM, Thermo Scientific Apreo2, Waltham, MA, USA) equipped with an energy dispersive spectrometer (EDS, OXFORD Ultim Max 40, Singapore). The phase compositions were identified by X-ray diffraction (XRD, Smartlab 9 (Boston, MA, USA), Cu Kα, λ = 1.5406 Å). The melting behavior of AgCuTi foil was evaluated by differential scanning calorimetry (DSC, NETZSCH DSC404F3) (Selb, Germany) in an argon atmosphere at a heating rate of 10 °C min^−1^. The Vickers hardness of the samples was measured with a load of 500 g and a dwell time of 10 s, and the average value was calculated from at least five measurements. To measure the shear strength of the sapphire/TC4 joints at room temperature, a custom-designed testing device (Figure 2b) was attached to a universal testing machine (EUTM, SHIMADZU AG-Xplus100KN, Kyoto, Japan). The displacement rate was set at 0.5 mm/min and the shear strength value was obtained from the average of at least five trials. The fracture surfaces were observed by a stereo microscope (SZX7, Olympus, Tokyo, Japan). The defects in the sapphire/TC4 joints were detected by a scanning acoustic microscope (KSI V400E, Herborn, Germany). The bulk density of the samples was measured by Archimedes’ method.

## 3. Results and Discussion

### 3.1. Microstructure and Melting Characteristics of the AgCuTi Foil

**Figure 3:**
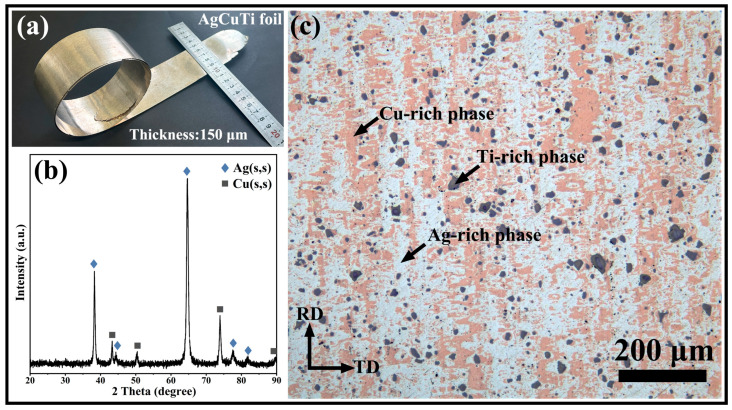
(**a**) Photograph of the AgCuTi foil showing its dimensions and smooth surface. (**b**) X-ray diffraction (XRD) pattern of the AgCuTi foil, indicating the presence of Ag solid solution (Ag(s,s)) and Cu solid solution (Cu(s,s)) phases. (**c**) Optical metallography image of the AgCuTi foil, revealing the microstructure. The white, brown, and gray zones correspond to the Ag-rich, Cu-rich, and Ti-rich phases, respectively. RD and TD denote the rolling and transverse directions, respectively.

A smooth AgCuTi foil with a thickness of about 150 μm was prepared by cold rolling (Figure 3a). The foil had a density of 9.61 g·cm^−3^ and a hardness of 183.5 HV. X-ray diffraction (XRD) showed only Ag(s,s) and Cu(s,s) peaks, indicating that the titanium and intermetallic compound (IMC) contents were too low to be detected by XRD (Figure 3b). Moreover, a discernible shift in the diffraction peak positions was observed for both Ag(s,s) and Cu(s,s) phases, as corroborated by the data presented in Figure S1. Optical metallography revealed the microstructure and phase distribution of the AgCuTi foil. As shown in Figure 3c, the white, brown, and gray zones represented the Ag-rich, Cu-rich, and Ti-rich phases, respectively. The Ag-rich and Cu-rich phases underwent deformation and elongation along the rolling direction, resulting in a finer and more uniform microstructure. The Ti-rich phases remained undeformed and unelongated along the rolling direction.

**Figure 4:**
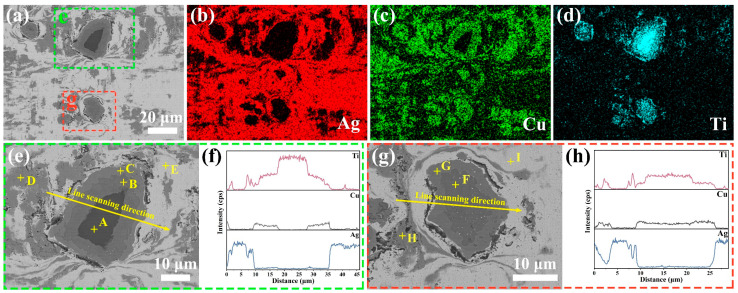
EDS mapping reveals the presence and relative abundance of Ag, Cu, and Ti elements in the foil (**a**–**d**). The regions enclosed by green and red dashed rectangles in (**a**) are enlarged in (**e**,**g**), respectively, showing the layered microstructure of Ti-rich phases. The corresponding EDS line scans along the yellow lines in (**e**,**g**) are presented in (**f**,**h**), respectively, indicating the variation of elemental concentrations across the Ti-rich phases.

FESEM-EDS analysis was employed to further characterize the phase composition of AgCuTi foil. The results of the EDS-mapping analysis confirmed the existence and distribution of Ag-rich, Cu-rich, and Ti-rich phases in the foil (Figure 4a–d). These phases were clearly distinguishable from each other. Notably, two kinds of Ti-rich phases were detected. One type had a Ti core (Figure 4e), while the other type did not (Figure 4g). Both types of Ti-rich phases showed a multi-layered structure as a result of the solid diffusion reaction. This layered microstructure was also verified by the EDS elemental line scans across the Ti-rich phases (Figure 4f,h).

**Figure 5:**
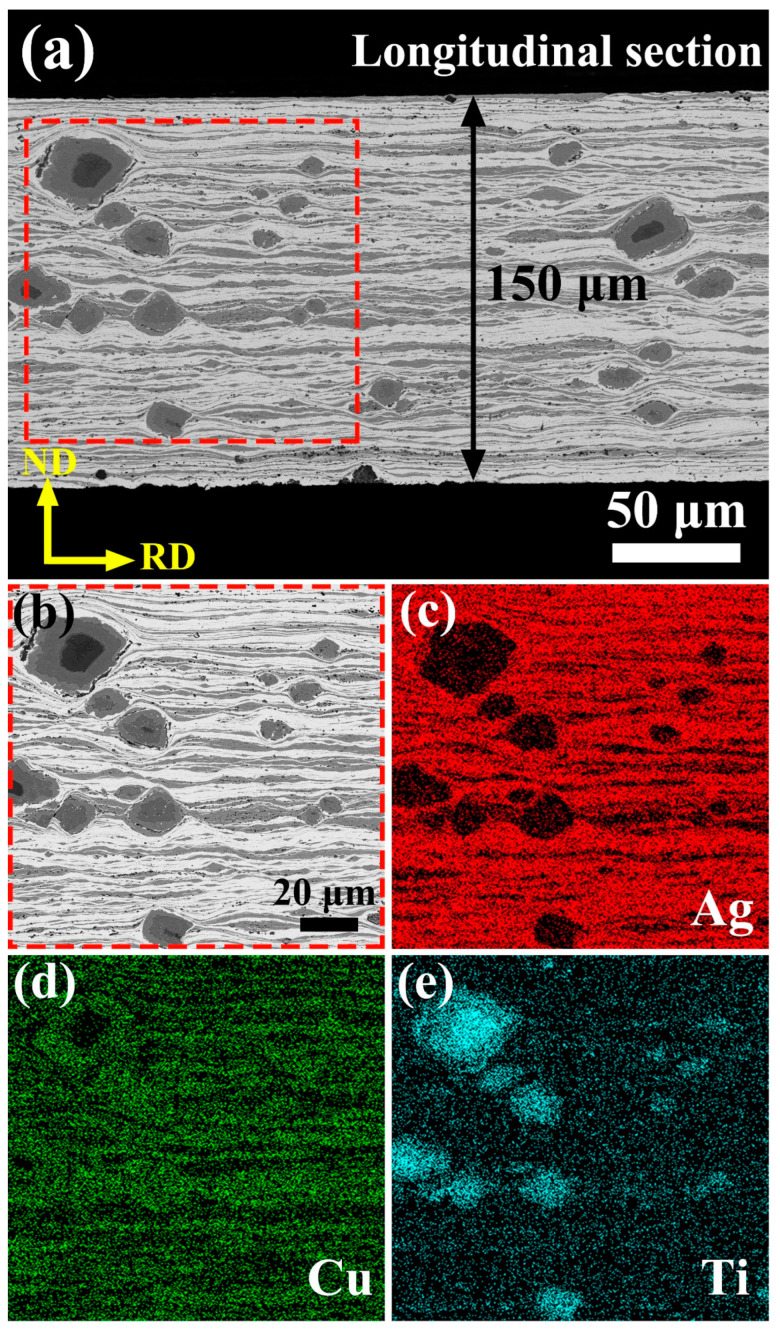
(**a**) Longitudinal section of the AgCuTi foil, showing its thickness and microstructural features. (**b**) EDS elemental maps of the area enclosed by the red dashed rectangle in (**a**), showing the distribution of Ag (**c**), Cu (**d**), and Ti (**e**) in the foil. RD and ND denote the rolling and normal directions, respectively.

The microstructure and phase composition of AgCuTi foil were further examined by FESEM-EDS analysis of its longitudinal section. The thickness of the foil was around 150 μm and its microstructural features are shown in Figure 5a. EDS elemental maps revealed the spatial distribution of Ag (Figure 5c), Cu (Figure 5d), and Ti (Figure 5e) in the foil, showing a clear separation of Ag-rich, Cu-rich, and Ti-rich phases. The Ag-rich and Cu-rich phases were elongated, while the layered Ti-rich phases, with or without a Ti core, remained undeformed in the foil.

To identify the composition of each phase, EDS spot analysis was conducted, and the results are shown in Table 1. Ti was the predominant element in spot A, which corresponded to the core of a layered Ti-rich phase. Spots B and F had comparable amounts of Cu and Ti, indicating TiCu phases. The Cu/Ti atomic ratio at spots C and G matched the stoichiometry of TiCu_4_. Spots D and H comprised mostly Cu and minor Ag, implying Cu(s,s) phases. Spots E and I consisted of mainly Ag and trace Cu, suggesting Ag(s,s) phases. The presence of TiCu and TiCu_4_ reflected the high chemical affinity of Ti and Cu [25,26]. Notably, no oxidation phase, particularly for Ti, was observed in the AgCuTi foil, demonstrating its potential for high brazing performance.

The novel microstructure observed in the AgCuTi foil is distinct from the conventional AgCuTi brazing foils Cusil ABA^®^ and Ticusil^®^, produced by different methods. The Ag-Cu eutectic alloy in Cusil ABA^®^ contains TiCu_4_ particles, resulting from melting, casting, and rolling [21]. Ticusil^®^ is produced by pack rolling AgCu/Ti/AgCu sheets and forms Ti_3_Cu_4_ layers adjacent to the Ti sheet upon heating [22]. The AgCuTi foil, however, exhibits a phase separation of Ag-rich and Cu-rich regions, and a multi-layered structure of ordered TiCu and TiCu_4_ phases in the Ti-rich regions, with or without a Ti core. This special microstructure could enhance the melting characteristics and brazing performance of AgCuTi foil.

**Figure 6:**
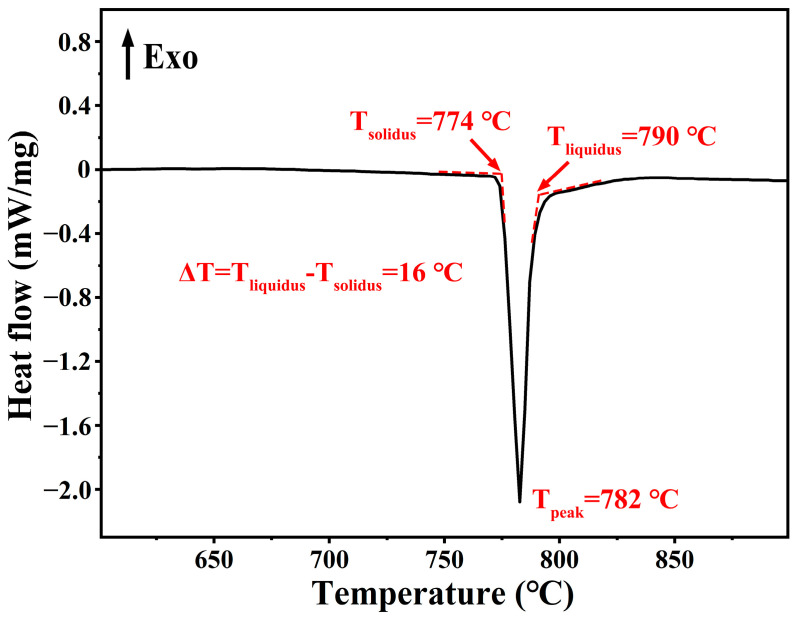
Melting behavior of AgCuTi brazing foil. The DSC curve exhibits an endothermic peak, indicating the melting of the AgCuTi alloy. The temperatures corresponding to the onset (T_solidus_), peak (T_peak_), and end (T_liquidus_) of the melting process are marked. The melting range (ΔT) is obtained by subtracting T_solidus_ from T_liquidus_.

The AgCuTi foil possesses a unique microstructure that confers its excellent melting properties. As shown in Figure 6, the differential scanning calorimetry (DSC) curve of the AgCuTi foil reveals a single endothermic peak, corresponding to the melting of the alloy. The solidus and liquidus temperatures (T_solidus_ and T_liquidus_) are 774 °C and 790 °C, respectively, resulting in a narrow melting range of 16 °C. The melting characteristics of a brazing alloy are critical for its performance, as they influence the flow and wetting of the molten metal [27]. A narrow melting range facilitates rapid and low-temperature brazing, whereas a wide melting range can lead to liquation and segregation of alloy components, compromising the joint quality. Compared with commercial brazing alloys such as Cusil ABA^®^ and Ticusil^®^, which have melting ranges of 35 °C and 120 °C, and liquidus temperatures of 815 °C and 900 °C, respectively, the AgCuTi foil exhibits superior melting behavior [21,22]. The sharp and narrow endothermic peak indicates the fast melting of the AgCuTi foil. These results suggest that the AgCuTi foil is a promising brazing material.

### 3.2. Microstructure of the Sapphire/TC4 Joint

**Figure 7:**
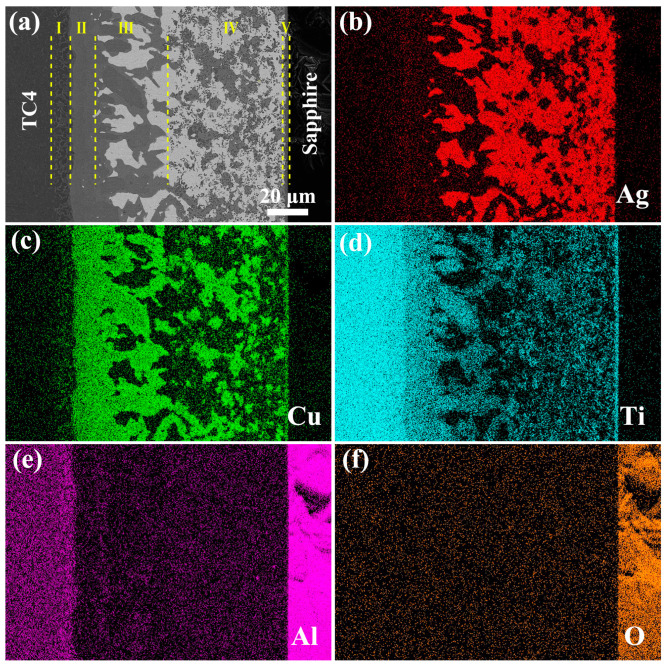
Microstructural characterization of the sapphire/TC4 brazed joint. (**a**) Backscattered electron image (BSEI) of the joint cross-section brazed at 800 °C for 10 min, showing five regions with different microstructures. (**b**–**f**) Energy-dispersive X-ray spectroscopy (EDS) elemental maps of Ag (**b**), Cu (**c**), Ti (**d**), Al (**e**), and O (**f**), showing the spatial distribution and chemical composition of the reaction phases in the joint.

**Figure 8:**
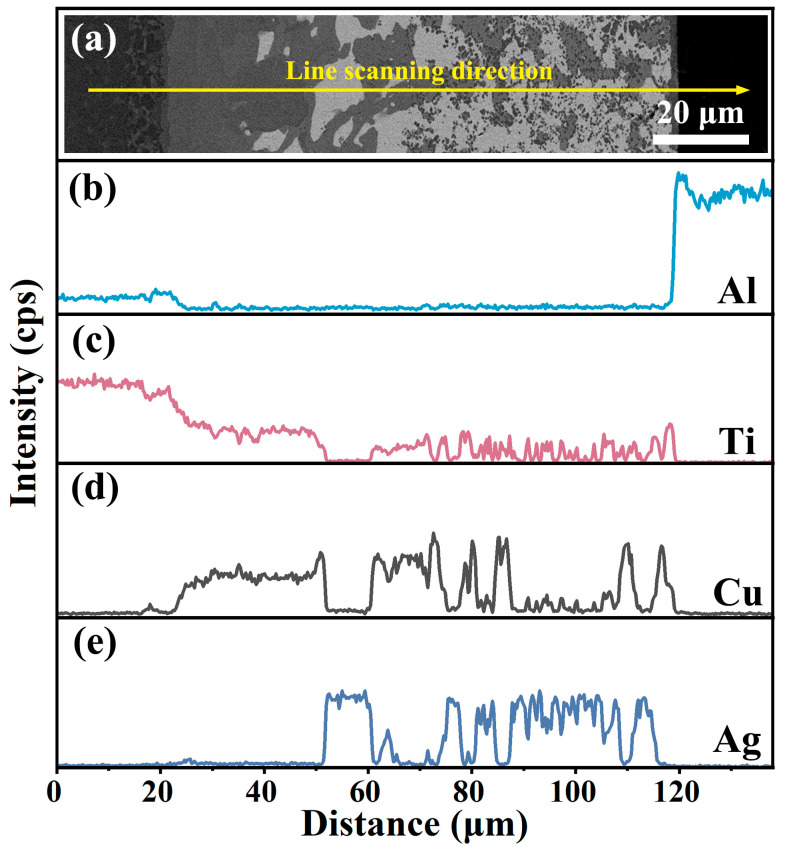
(**a**) Backscattered electron image (BSEI) of the joint, with the EDS line scan (yellow line) showing the direction and position of the elemental analysis. (**b**–**e**) EDS line profiles of Al (**b**), Ti (**c**), Cu (**d**), and Ag (**e**), revealing the concentration gradients of elements.

The brazing filler of AgCuTi foil was employed to bond TC4 and sapphire at 800 °C for 10 min. The microstructure of the joint, shown in Figure 7a, was free of cracks or voids. EDS-mapping (Figure 7b–f) and EDS line scan analysis (Figure 8) revealed the extensive formation of Ti-Cu intermetallic compounds (IMCs) in the joint. These IMCs originated from the preferential reaction and dissolution of Ti in Cu rather than in Ag, due to their higher chemical affinity and solubility. The Ti content varied from high on the TC4 side to low on the sapphire side. Cu was mainly present in Ti-Cu IMCs and Cu(s,s), while Ag was confined to Ag(s,s). A small amount of Al was found in the reaction layer near the sapphire.

**Figure 9:**
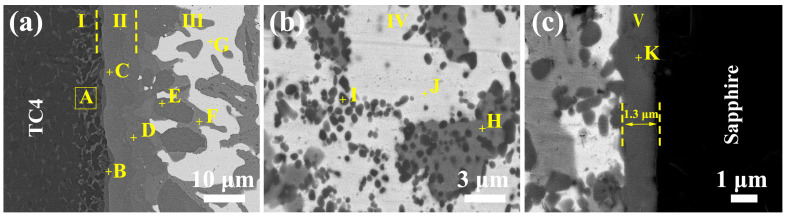
(**a**–**c**) Backscattered electron (BSE) images at high magnification reveal the phase composition of the five regions shown in Figure 7. The phases, identified by energy-dispersive X-ray spectroscopy (EDS) and labeled from A to K, are listed in Table 2.

**Figure 10:**
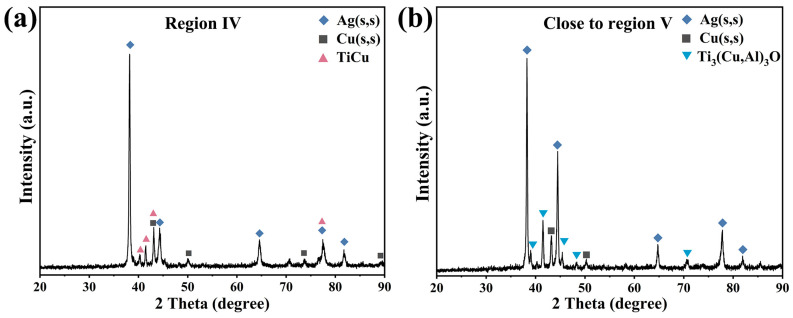
(**a**) XRD pattern of region IV shown in Figure 7, indicating the formation of Ag solid solution (Ag(s,s)), Cu solid solution (Cu(s,s)), and TiCu phases. (**b**) XRD pattern of a region near region V (Figure 7), revealing the existence of Ag(s,s), Cu(s,s), and Ti_3_(Cu,Al)_3_O phases. The joint was ground and polished to expose the test region.

As depicted in Figure 7 and Figure 9, the sapphire/TC4 joint has a heterogeneous microstructure with five different regions. Region I is the TC4 matrix with a coarse α-β Ti microstructure, resulting from the β-Ti stabilizing effect of Cu and Ag that reduces the β→α transformation temperature of Ti [28,29]. After brazing and cooling, more β-Ti remains in the microstructure, appearing bright, while α-Ti appears dark. Regions II, III, and IV contain various Ti-Cu intermetallic compounds (IMCs) (Ti_2_Cu, Ti_3_Cu_4_, Ti_2_Cu_3_, TiCu, and TiCu_4_), identified by their composition and the Ti-Cu phase diagram [30]. Ti_2_Cu, Ti_3_Cu_4_, and Ti_2_Cu_3_ form region II, whereas region III contains TiCu, TiCu_4_, and Ag(s,s). Region IV has a mixture of Ag(s,s), Cu(s,s), and fine TiCu granules (average size ~500 nm), which may increase the joint strength [31,32]. Region V is a thin (~1.3 μm) reaction layer next to the sapphire, with a dense and continuous Ti_3_(Cu,Al)_3_O phase that has a metallic nature and may enhance the wetting and bonding [33]. X-ray diffraction (XRD) analysis, as depicted in Figure 10, reveals the presence of Ag(s,s) and Cu(s,s), characterized by shifted diffraction peaks (Supplementary Figure S2). Additionally, the presence of TiCu is observed in region IV, while Ti_3_(Cu,Al)_3_O is identified in region V. The characteristic diffraction peaks corresponding to TiCu and Ti₃(Cu,Al)₃O align closely with those indexed in the Powder Diffraction File (PDF) entries numbered 07-0114 and 49-0665, respectively. No TiO_x_ layer is observed adjacent to the sapphire, possibly because it is unstable below 1250 °C [34,35].

### 3.3. Non-Destructive Evaluation and Shear Strength of the Sapphire/TC4 Joint

**Figure 11:**
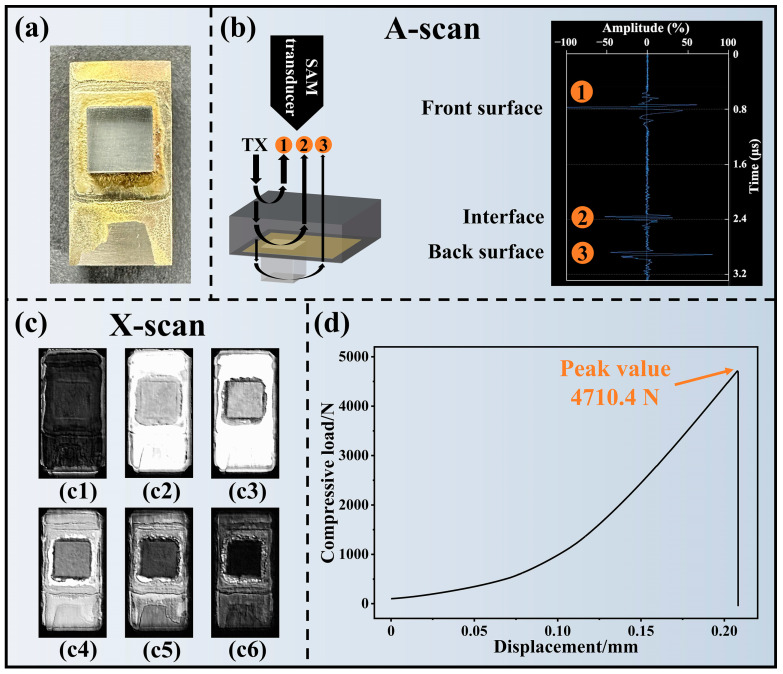
(**a**) Optical image of the sapphire/TC4 joint. (**b**) A-scan scanning acoustic microscopy (SAM) image of the acoustic impedance across the joint. (**c**) X-scan SAM image of the joint, with (**c1**–**c6**) showing the microstructure and defects. (**d**) Compressive load–displacement curve of the joint, demonstrating its mechanical strength.

Using AgCuTi brazing foil as the filler metal, a defect-free and intact sapphire/TC4 joint (Figure 11a) was achieved. The sapphire showed no signs of damage or cracking. The fillet formation on the TC4 surface outside the interface indicated the good fluidity and wettability of the brazing alloy.

The integrity of the sapphire/TC4 joint was evaluated by scanning acoustic microscopy (SAM) in A-scan and X-scan modes (Figure 11b,c). The A-scan mode revealed the front surface, interface, and back surface of the joint (Figure 11b), while the X-scan mode generated 2D images of the target layers at various depths in the joint (Figure 11c). The SAM images showed no signs of defects, such as delamination or voids, in TC4, brazing seam, or sapphire, suggesting a strong bond between the sapphire and the TC4 alloy.

**Figure 12:**
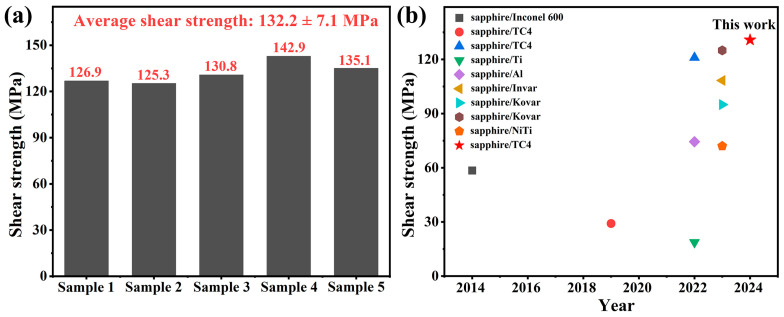
(**a**) The shear strength of the sapphire/TC4 joints for samples 1–5 is shown, with a mean value of 132.2 ± 7.1 MPa. (**b**) A comparison of the mean shear strength of the sapphire/TC4 joints and other sapphire/metal joints reported in the literature [23,32,36,37,38,39,40,41,42].

The shear strength of the sapphire/TC4 joint was measured by applying a load until a sudden fracture occurred. Figure 11d illustrates one of the compressive load–displacement curves. The maximum load was 4710.4 N, which corresponded to a shear strength of 130.8 MPa. Figure 12 and Table 3 compare the average shear strength of 132.2 MPa with the shear strength of sapphire/metal joints from various existing studies. The sapphire/TC4 brazed joint exhibited the highest shear strength among all the reported studies, indicating a superior mechanical performance.

### 3.4. Fracture Features of Sapphire/TC4 Joint under Shear Stress

**Figure 13:**
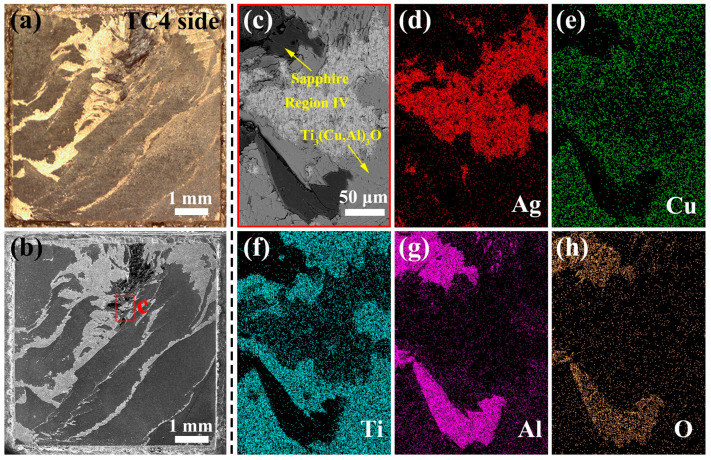
Characterization of the fracture surface on the TC4 side of the sapphire/TC4 joint. (**a**) Optical micrograph and (**b**) SE-SEM image of the fracture surface, revealing its morphology. (**c**) High-magnification BSE-SEM image of the region marked by the red rectangle in (**b**). (**d**–**h**) EDS maps show the distribution of Ag (**d**), Cu (**e**), Ti (**f**), Al (**g**), and O (**h**) in (**c**).

To elucidate the fracture mechanism of the sapphire/TC4 joint, the fracture surfaces on the TC4 side were examined by fractography analysis (Figure 13a,b). These surfaces were expected to display a brittle fracture mode according to the compressive load–displacement curve. The fractography analysis revealed large areas of Ti_3_(Cu,Al)_3_O (reaction layer, region V), small areas of region IV (Ag(s,s), Cu(s,s), and TiCu granules), and remnants of sapphire, as verified by EDS mapping analysis (Figure 13c–h). The fracture surfaces exhibited an elemental distribution that matched the joint microstructure shown in Figure 7. On the other hand, the sapphire side fractured into multiple pieces.

The fracture surface provides evidence for the joint fracture mechanism, which exhibits two characteristics: (i) partial exposure of regions IV and V, where region V is the Ti_3_(Cu,Al)_3_O reaction layer; and (ii) prevalent fracture at the interface between region V and sapphire. The conventional adhesive failure mode cannot account for the presence of region IV and sapphire on the fracture surface. Therefore, a more complex fracture mechanism is probable, which necessitates further research.

The fracture surface exhibits two possible sites for crack initiation and propagation, namely the interface between sapphire and region V, and the interior of region IV. Depending on the site of crack initiation, different types of dimples can be observed in region IV on the fracture surface. Tearing dimples indicate that the crack originated from the sapphire–region V interface and propagated into region IV, while shear dimples imply that the crack originated from the interior of region IV. Therefore, the dimple morphology in region IV on the fracture surface provides insights into the crack initiation and propagation mechanisms.

**Figure 14:**
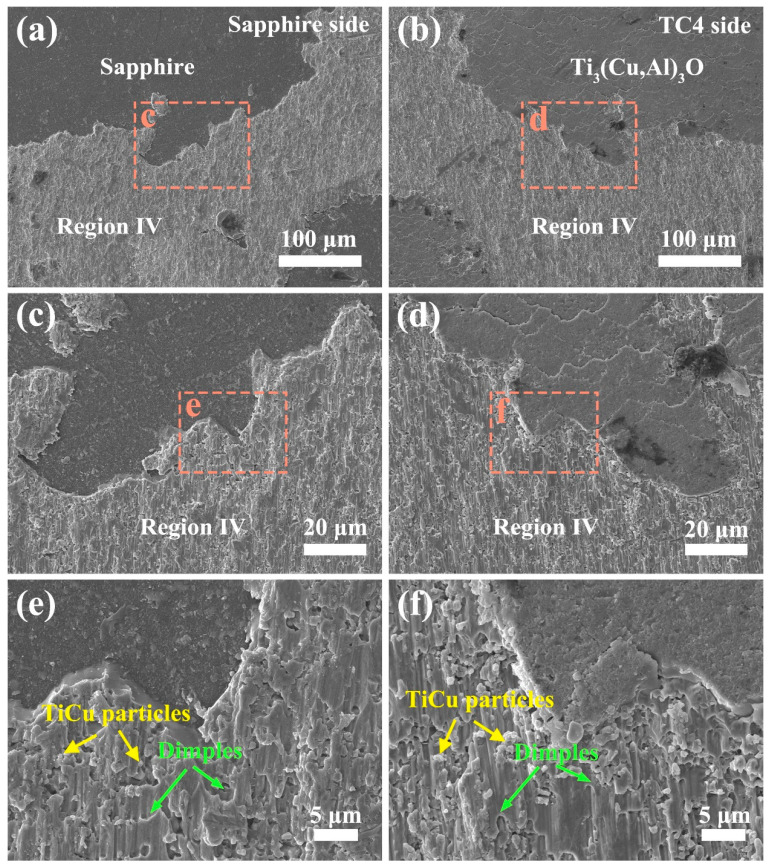
The fracture surfaces of the sapphire (**a**,**c**,**e**) and TC4 (**b**,**d**,**f**) sides are displayed in matching pairs. The areas marked by orange dotted rectangles are magnified in the subsequent images. Green arrows point to the elongated dimples and their opening direction. Yellow arrows indicate the presence of TiCu particles in region IV.

The dimple orientation on the matching fracture surfaces can differentiate shear and tearing dimples. Shear dimples have opposite orientations while tearing dimples have the same orientation [43]. Figure 14 shows that the matching fracture surfaces exhibit opposite-oriented elongated dimples, indicating shear dimples. This implies that cracks initiate and propagate in region IV, according to the previous analysis. The cracks then breach the reaction layer and advance along the reaction layer–sapphire interface, causing the sapphire/TC4 joint to fracture brittlely. Thus, region IV splits into two parts, which appear on both fracture surfaces; the TC4-side fracture surface has large areas of Ti_3_(Cu,Al)_3_O.

**Figure 15:**
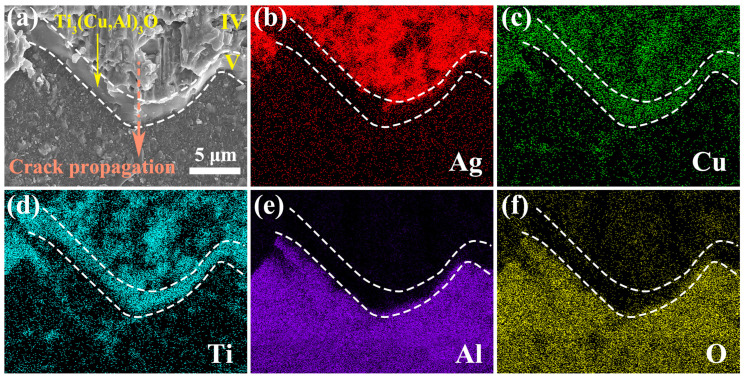
(**a**) A high-magnification SE-SEM image of the fracture surface on the sapphire side, revealing three distinct regions (region IV, region V, and the sapphire/region V interface that separated during fracture) along the crack path. The orange arrow indicates the direction of crack propagation. (**b**–**f**) EDS maps of the same area as in (**a**), showing the spatial distribution of Ag (**b**), Cu (**c**), Ti (**d**), Al (**e**), and O (**f**) elements.

**Figure 16:**
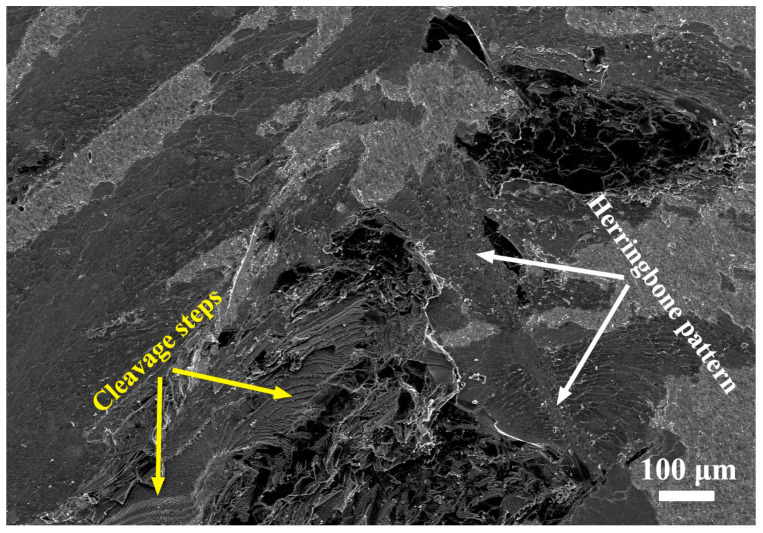
Fracture characteristics of sapphire and the reaction layer Ti_3_(Cu,Al)_3_O. The sapphire crystal reveals distinct cleavage steps, while the reaction layer of Ti_3_(Cu,Al)_3_O exhibits a distinctive herringbone pattern.

The high-magnification SEM image in Figure 15 illustrates the fracture surface on the sapphire side, which comprises three different regions (region IV, region V, and the sapphire/region V interface) along the crack path. Region V corresponds to the reaction layer of Ti_3_(Cu,Al)_3_O, as verified by the EDS mapping analysis in Figure 15b–f, which displays the distribution and relation of region IV, region V, and sapphire on the fracture surface. The fracture surface of region V is notably smooth and links the shear dimples in region IV and the interfaces between region V and sapphire. This implies the crack propagation from region IV to the interface between the reaction layer and sapphire. The crack may have deviated at this interface, resulting in a slightly rougher interface exposed on the fracture surface (Figure 15a). The crack may have also intruded into the sapphire, leading to its fragmentation. Consequently, some sapphire pieces are visible on the fracture surface of the TC4 side (Figure 13 and Figure 16).

Figure 16 shows the brittle fracture features of sapphire and the reaction layer Ti_3_(Cu,Al)_3_O. A brittle fracture of the Ti_3_(Cu,Al)_3_O layer is indicated by its remarkably smooth fracture surface (Figure 15). This is further confirmed by the herringbone pattern observed on the fracture surface of the Ti_3_(Cu,Al)_3_O layer (Figure 16), which is a characteristic feature of cleavage fracture. The sapphire fracture surface also exhibits cleavage fracture mode, as evidenced by the multiple cleavage steps (Figure 16). The cleavage fracture of both the Ti_3_(Cu,Al)_3_O and sapphire led to the sudden brittle fracture of the sapphire/TC4 joint (Figure 11d).

### 3.5. Fracture Mechanism of Sapphire/TC4 Joint under the Shear Stress

**Figure 17:**
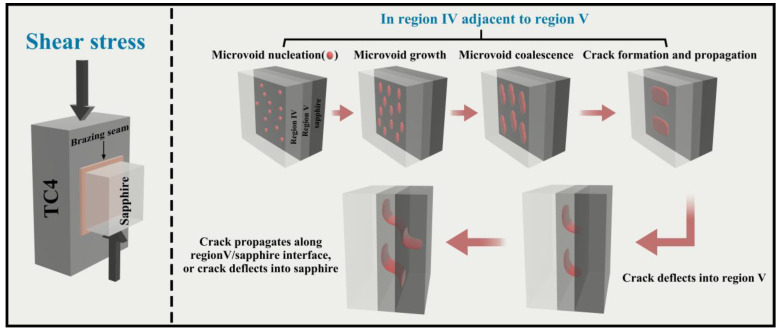
Schematic illustration of crack initiation and propagation in a sapphire/TC4 joint.

Figure 17 illustrates the critical processes involved in crack initiation and subsequent propagation within a sapphire/TC4 joint. The strain discontinuity observed in region IV leads to the nucleation of microvoids under shear stress. As the strain continues to increase, these microvoids grow, coalesce, and eventually form elongated shear dimples and continuous fracture surfaces within the same region (as depicted in Figure 14). Subsequently, the crack deviates into the reaction layer, resulting in cleavage fracture and the generation of smooth fracture surfaces composed of the Ti_3_(Cu,Al)_3_O reaction layer (as shown in Figure 15).

The crack then proceeds to propagate along the interface between the Ti_3_(Cu,Al)_3_O reaction layer and the sapphire material. During this propagation, certain portions of the crack detach the sapphire and the reaction layer, leaving a slightly rougher interface on the fracture surface (Figure 15). Simultaneously, other segments of the crack deflect into the sapphire, leading to cleavage fracture specifically within the sapphire material (as depicted in Figure 16).

Notably, cleavage fracture typically occurs at an exceptionally high speed. The rapid cleavage fracture of the reaction layer and sapphire ultimately results in the sudden brittle fracture of the sapphire/TC4 joint (as observed in Figure 11d). Consequently, one potential avenue for enhancing ceramic/metal joints lies in inhibiting crack initiation and propagation specifically within region IV. This comprehensive understanding of the fracture mechanism in sapphire/TC4 joints serves as a robust foundation for the development of high-strength and reliable ceramic/metal joint designs.

## 4. Conclusions

We have developed a novel AgCuTi brazing foil with a special structure that enables strong and defect-free bonding between sapphire and TC4 alloy. Our main findings are:

(1) The AgCuTi brazing foil, with a thickness of 150 μm, displayed a distinctive microstructure comprising solid solutions of Ag and Cu, as well as layered Ti-rich phases. This unique microstructure was responsible for the exceptional thermal properties of the AgCuTi brazing foil, including a low liquidus temperature of 790 °C and a remarkably narrow melting range of 16 °C, which surpassed the performance of previously reported AgCuTi brazing materials.
(2) A defect-free sapphire/TC4 joint was successfully fabricated via vacuum brazing using the 150 μm thick AgCuTi foil filler, which exhibited a heterogeneous microstructure comprising five distinct regions with unique phase compositions. The resulting joint demonstrated a shear strength of up to 132.2 MPa, which was attributed to the synergistic effects of the various phase compositions present in each region.
(3) Crack nucleation was predominantly observed within region IV, subsequently propagating through the reaction layer via a cleavage mechanism. This process either culminated in interfacial debonding or penetrated into the sapphire. The fracture surfaces of both the Ti_3_(Cu,Al)_3_O reaction layer and the sapphire displayed characteristic cleavage morphologies. The brittle fracture of the sapphire/TC4 joint is therefore ascribed to the combined influence of cleavage failure within both the reaction layer and the sapphire.

## Figures and Tables

**Table 1:** Composition (at.%) and possible phase of spots marked in Figure 4e,g.

EDS Spot	Ag	Cu	Ti	Possible Phase
Figure 4e, point A	-	0.91	99.09	Ti
Figure 4e, point B	0.17	49.33	50.50	TiCu
Figure 4e, point C	1.25	77.43	21.32	TiCu_4_
Figure 4e, point D	7.25	92.75	-	Cu(s,s)
Figure 4e, point E	93.26	6.74	-	Ag(s,s)
Figure 4g, point F	2.19	52.69	45.12	TiCu
Figure 4g, point G	0.64	78.99	20.37	TiCu_4_
Figure 4g, point H	8.44	87.82	3.74	Cu(s,s)
Figure 4g, point I	92.79	7.21	-	Ag(s,s)

**Table 2:** Composition (at.%) and possible phase of spots marked in Figure 9.

EDS Spot	Ag	Cu	Ti	Al	V	O	Possible Phase	Region
Figure 9a, point A	0.53	3.93	67.61	8.88	13.37	5.68	α-β Ti	I
Figure 9a, point B	1.78	29.07	62.93	5.17	1.05	-	Ti_2_Cu	II
Figure 9a, point C	1.56	55.79	42.52	0.13	-	-	Ti_3_Cu_4_	II
Figure 9a, point D	1.25	58.23	37.78	2.74	-	-	Ti_2_Cu_3_	II
Figure 9a, point E	1.31	46.34	45.68	0.59	1.79	4.29	TiCu	III
Figure 9a, point F	1.62	77.53	20.85	-	-	-	TiCu_4_	III
Figure 9a, point G	89.59	10.41	-	-	-	-	Ag(s,s)	III
Figure 9b, point H	2.88	94.26	2.86	-	-	-	Cu(s,s)	IV
Figure 9b, point I	1.46	52.44	45.55	0.55	-	-	TiCu	IV
Figure 9b, point J	91.26	8.74	-	-	-	-	Ag(s,s)	IV
Figure 9c, point K	0.30	26.20	44.95	11.01	0.84	16.70	Ti_3_(Cu,Al)_3_O	V

**Table 3:** Shear strength of sapphire/TC4 and other sapphire/metal joints.

System	Joining Technique	Shear Strength	Year	Ref.
Sapphire/Inconel 600	Brazing	58.5 MPa	2014	[36]
Sapphire/TC4	Brazing	29.1 MPa	2019	[37]
Sapphire/TC4	Diffusion bonding	121 MPa	2022	[38]
Sapphire/Ti	Brazing	18.7 MPa	2022	[39]
Sapphire/Al	Ultrasonic soldered	74.42 MPa	2022	[40]
Sapphire/Invar	Femtosecond laser micro-welding	108.35 MPa	2023	[41]
Sapphire/Kovar	Brazing	95 MPa	2023	[23]
Sapphire/Kovar	Brazing	125 MPa	2023	[32]
Sapphire/NiTi	Transient liquid phase bonding	72 MPa	2023	[42]
Sapphire/TC4	Brazing	132.2 MPa	2024	This work

## Data Availability

The original contributions presented in the study are included in the article/Supplementary Material, further inquiries can be directed to the corresponding author.

## Supplementary Materials

The following supporting information can be downloaded at: https://www.mdpi.com/article/10.3390/ma17153812/s1, Figure S1: Peak shift analysis of Ag(s,s) and Cu(s,s) diffraction patterns; Figure S2: A pronounced shift in the diffraction peak positions of Ag(s,s) and Cu(s,s) was observed in the XRD patterns. (a) XRD pattern of region IV. (b) XRD pattern of the region adjacent to region V.
