# Supplementary material for: Specially Structured AgCuTi Foil Enables High-Strength and Defect-Free Brazing of Sapphire and Ti6Al4V Alloys: The Microstructure and Fracture Characteristics
Source: Materials (Basel). 2024 Aug 2;17(15):3812. doi: 10.3390/ma17153812 (PMC11313281; doi:10.3390/ma17153812)
Supplement: Supplementary file 1 [file materials-17-03812-s001.zip › materials-3066508-supplementary.pdf]

Supplementary Information to:

Specially structured AgCuTi foil enables high-strength and defect-free brazing of sapphire and Ti6Al4V alloys: the microstructure and fracture characteristics

Shaohong Liu [1,2,*], Hairui Liu [1], Limin Zhou [2], Hao Cui [2], Manmen Liu [2,3], Li Chen [2], Ming Wen [2], Haigang Dong [2], Feng Liu [2], Wei Wang [1] and Song Li [1]

1 Key Laboratory for Anisotropy and Texture of Materials (Ministry of Education), School of Materials Science and Engineering, Northeastern University, Shenyang 110819, China; lhairui@stumail.neu.edu.cn (H.L.); wangw@atm.neu.edu.cn (W.W.); lis@atm.neu.edu.cn (S.L.)
2 State Key Laboratory of Advanced Technologies for Comprehensive Utilization of Platinum Metals, Yunnan Precious Metals Laboratory Co., Ltd., Kunming 650106, China; zlm@ipm.com.cn (L.Z.); cuihao@ipm.com.cn (H.C.); lmm@ipm.com.cn (M.L.); chenli@ipm.com.cne (L.C.); wen@ipm.com.cn (M.W.); dhg@ipm.com.cne (H.D.); liufeng@ipm.com.cne (F.L.)
3 Sino-Platinum Metals Semiconductor Materials (Yunnan) Co., Ltd., Kunming 650106, China
* Correspondence: liush@smm.neu.edu.cn; Tel.: +86-13604025172

Figure S1 elucidates the observed shifts in diffraction peaks for Ag(s,s) and Cu(s,s). Specifically, the diffraction peaks associated with Ag(s,s) exhibit a displacement toward higher angles, while those corresponding to Cu(s,s) demonstrate a shift toward lower angles.

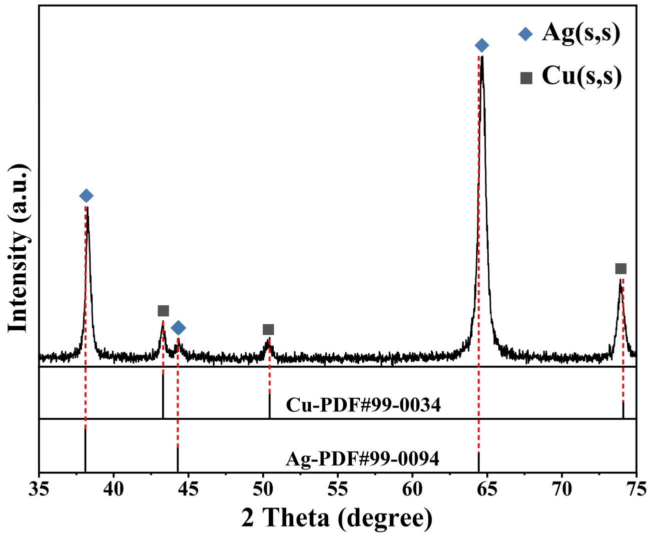

Figure S1. Peak shift analysis of Ag(s,s) and Cu(s,s) diffraction patterns.

Figure S2 presents the X-ray diffraction (XRD) patterns of region IV and an adjacent region proximate to region V. Notably, a shift in diffraction peaks was observed for Ag(s,s) towards higher angles and Cu(s,s) towards lower angles, indicating potential lattice parameter variations.

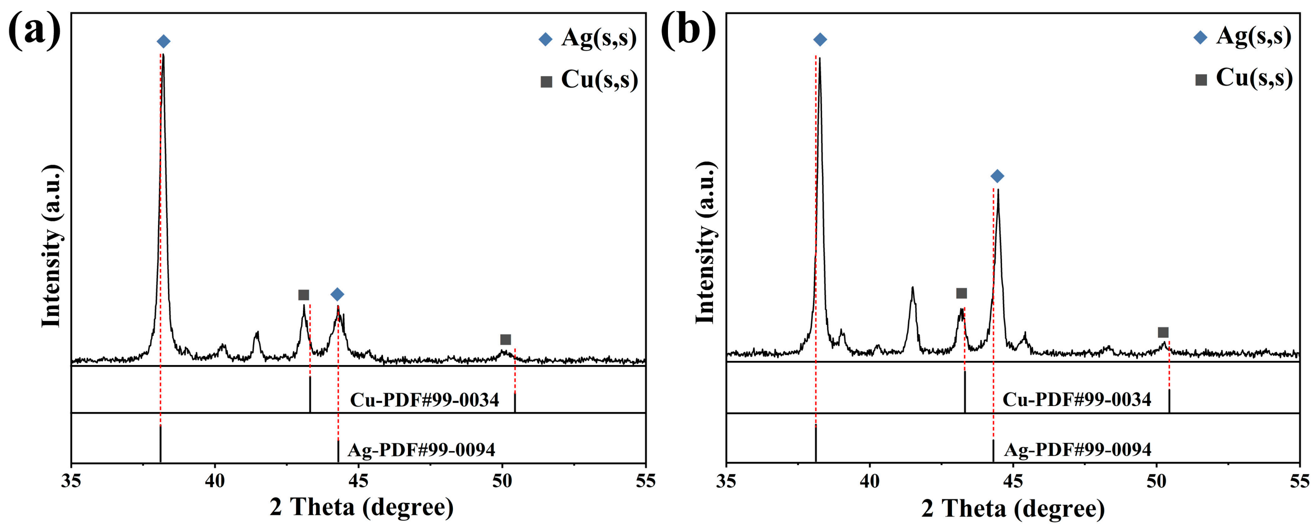

Figure S2. A pronounced shift in the diffraction peak positions of Ag(s,s) and Cu(s,s) was observed in the XRD patterns. (a) XRD pattern of region IV. (b) XRD pattern of the region adjacent to region V.
